# Green Synthesis of Nickel Oxide Nanoparticles from *Berberis balochistanica* Stem for Investigating Bioactivities

**DOI:** 10.3390/molecules26061548

**Published:** 2021-03-11

**Authors:** Siraj Uddin, Luqman Bin Safdar, Saeed Anwar, Javed Iqbal, Sabiha Laila, Banzeer Ahsan Abbasi, Muhammad Saqib Saif, Musrat Ali, Abdul Rehman, Abdul Basit, Yong Wang, Umar Masood Quraishi

**Affiliations:** 1 Department of Plant Sciences, Faculty of Biological Sciences, Quaid-i-Azam University, Islamabad 45320, Pakistan; usiraj@bs.qau.edu.pk (S.U.); saeedanwar@bs.qau.edu.pk (S.A.); jiqbal@bs.qau.edu.pk (J.I.); banzeer.abbasi@bs.qau.edu.pk (B.A.A.); musrat.ali@bs.qau.edu.pk (M.A.); 2 Plant Breeding Institute, University of Sydney, Narellan, NSW 2567, Australia; 3 School of Biosciences, Sutton Bonington Campus, University of Nottingham, Sutton Bonington, Leicestershire LE12 5RD, UK; 4 School of Agriculture, Food and Wine, Waite Research Institute, University of Adelaide, Glen Osmond, SA 5064, Australia; 5 Department of Botany, Bacha Khan University, Charsadda 24420, Pakistan; 6 Department of Botany, Sardar Bahadur Khan Women’s University, Quetta 1800, Pakistan; sabiha.qumber786@gmail.com; 7 Department of Biochemistry, The Islamia University of Bahawalpur, Bahawalpur 63100, Pakistan; saqibsaif.qau@gmail.com; 8 Centre of Excellence in Solid State Physics, Quaid I Azam Campus, University of Punjab, Lahore 54000, Pakistan; abdul.bsphy510@iiu.edu.pk; 9 Department of Plant Pathology, Agriculture College, Guizhou University, Guiyang 550025, China; basit@gzu.edu.cn

**Keywords:** NiONPs, green synthesis, antioxidants, antimicrobial, cytotoxicity, nano fertiliser

## Abstract

Green synthesis of nanomaterials is advancing due to its ease of synthesis, inexpensiveness, nontoxicity and renewability. In the present study, an eco-friendly biogenic method was developed for the green synthesis of nickel oxide nanoparticles (NiONPs) using phytochemically rich *Berberis balochistanica* stem (BBS) extract. The BBS extract was rich in phenolics, flavonoids and berberine. These phytochemicals successfully reduced and stabilised the NiNO_3_ (green) into NiONPs (greenish-gray). BBS-NiONPs were confirmed by using UV-visible spectroscopy (peak at 305 nm), X-ray diffraction (size of 31.44 nm), Fourier transform infrared spectroscopy (identified -OH group and Ni-O formation), energy dispersive spectroscopy (showed specified elemental nature) and scanning electron microscopy (showed rhombohedral agglomerated shape). BBS-NiONPs were exposed to multiple in vitro bioactivities to ascertain their beneficial biological applications. They exhibited strong antioxidant activities: total antioxidant capacity (64.77%) and 2, 2-diphenyl-1-picrylhydrazyl (71.48%); and cytotoxic potential: Brine shrimp cytotoxicity assay with IC_50_ (10.40 µg/mL). BBS-NiONPs restricted the bacterial and fungal pathogenic growths at 1000, 500 and 100 µg/mL. Additionally, BBS-NiONPs showed stimulatory efficacy by enhancing seed germination rate and seedling growth at 31.25 and 62.5 µg/mL. In aggregate, BBS extract has a potent antioxidant activity which makes the green biosynthesis of NiONPs easy, economical and safe. The biochemical potential of BBS-NiONPs can be useful in various biomedical and agricultural fields.

## 1. Introduction

Nanotechnology is revolutionising many industrial and technological fields due to the fact that it is possible with nanotech to orient material structures at extremely small scales, thereby extending the materials science toolkit. By using nanotechnology, materials can be made lighter, stronger, more reactive, better electrical conductors and more durable, among countless other characteristics. The properties of nanoparticles (NPs) can be altered by changing their size at the nanoscale which provides the capacity to use them in multidisciplinary fields including medicine [1]. Up until now, a variety of metals and metal oxide NPs have been synthesised such as silver, gold, platinum, magnesium, iron oxide, caesium oxide and zinc oxide [1,2]. Among these prepared NPs, nickel oxide NPs (NiONPs) have fascinated scientists from multiple fields due to their easy synthesis, small size, wide bandgap and semiconductor properties [3]. NiONPs have been reported with numerous fruitful applications in batteries, sensors, superconductors, magnetic materials, photocatalytic and catalytic analysis [4]. In green synthesis, three main sources, i.e., autotrophs (plant and algae), bacteria and fungi are used [3]. Plant-mediated green synthesis is getting significant value in biomedical fields due to its simplicity, easy availability, ecofriendly nature and nontoxicity, and it eradicates the prerequisite of reducing agents and energy from the external environment. Studies show that plants contain numerous valuable bioactive compounds like alkaloids, polyphenols, flavonoids, terpenoids, vitamins and minerals, which play a vital role as stabilising, capping and reducing agents during phytofabrication of NPs [5]. These phytochemicals also have significant antioxidant, anti-microbial, anti-inflammatory, chemo-preventative and cytotoxic potentials [6]. During green synthesis, phytochemicals with strong antioxidant potentials adsorb onto the NPs surface and make them effective antioxidant, antimicrobial, and cytotoxic NPs [7]. Therefore, the green synthesis of NPs serves as a useful and eco-friendly approach. The focus of green synthesis is being rapidly shifted toward medicinal plants due to their rich biological potential [8].

Plants belonging to the family Berberidaceae are catching interest to prepare NPs with great medical and phytonutritional properties, some recent examples are: [9,10,11]. The endemic shrub, *B. balochistanica* (Zralga), belongs to the family Berberidaceae and is found in the Quetta, Ziarat and Kalat regions of Balochistan, Pakistan [12]. The decoction of the underground part (root) is used for the treatment of various diseases like coughing, fever, wound healing, internal injury, eye disease, rheumatism and other infections of human beings and livestock [13,14,15,16,17]. Recently, the roots of this plant were studied and various secondary metabolites like berberisinol, berberine, palmatine, 8-oxoberberine, oleanolic acid, β-sitosterol, gallic acid, phenols, carotenoids and vitamins were isolated [13,17]. These secondary metabolites in the root were found with remarkable antioxidant, antibacterial and antifungal potentials [17,18]. The presence of bioactive compounds with antioxidative and antimicrobial properties makes this plant valuable to use for the green synthesis of NPs. However, there is little data on the chemical composition and antioxidant activity of *B. balochistanica* stem extract and its usage in green synthesis.

The aim of this study was to characterise the phytochemical and antioxidant activities of stem extract of *B. balochistanica*. After confirmation of biomolecules with significant antioxidant activities, the extract was subjected to synthesise NiONPs. The synthesised NiONPs were characterised using UV-visible, Fourier transform infrared (FTIR), energy dispersion spectroscopy (EDS), X-ray diffractometer (XRD) and scanning electron microscopy (SEM) analysis. To evaluate the biological potential of BBS-NiONPs, different in vitro biological and biofertiliser activities including antimicrobial, antioxidant, cytotoxic, inhibitory and biostimulatory activities were assessed.

## 2. Results

### 2.1. Physical and Morphological Characterisation of BBS-NiONPs 

**Figure 1:**
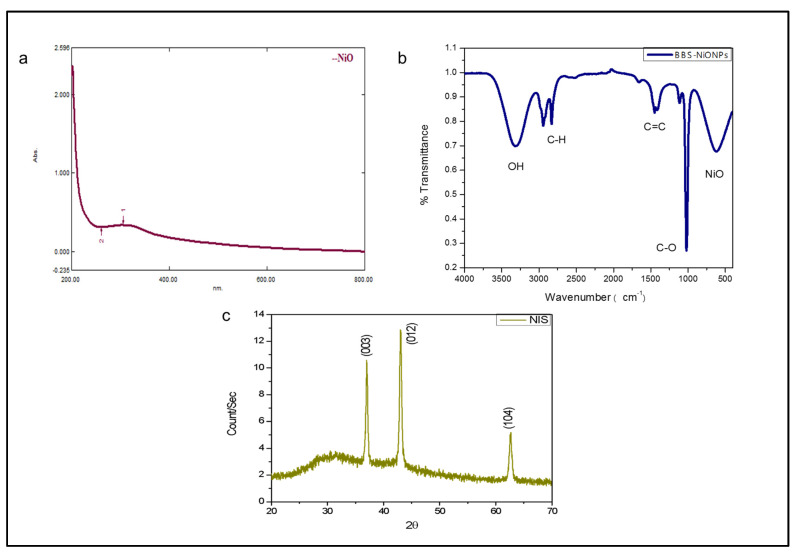
Spectroscopic profile of *Berberis balochistanica* stem (BBS)- nickel oxide nanoparticles (NiONPs). (**a**) UV-Vis spectrum of BBS-NiONPs. The peak value at 305.00 nm specifies the absorption of metal ions. (**b**) FTIR profile of BBS-NiONPs. Peaks indicate the presence of different functional groups. (**c**) XRD spectrum of BBS-NiONPs.

The confirmation of BBS-NiONPs was observed by various techniques and the results were validated by preceding analysis. Figure 1a represents the UV-Vis absorption spectrum of BBS-NiONPs formations using stem extract of *B. balochistanica.* The peak value at 305.00 nm specifies the absorption of metal ions. Figure 1b represents the FTIR spectra with multiple peaks belonging to bio-constituents adsorbed by the synthesised NPs. The FTIR profile of NiO displayed vibration at 3309.75 cm^−1^ for -OH groups, at 2943.78 and 2831.77 cm^−1^ indicating the C-H stretching, while other multiple peaks at 1559.94, 1449.04,1415.19,1114.62 and 1021.53 cm^−1^ were related to C=C and C-O stretching for aromatic ring and polyphenols. The peak at 616.36 cm^−1^ displayed information about NiO in NiNO_3_. The crystalline nature of BBS-NiONPs was analysed using the XRD technique. Sample NiONPs showed crystallite size (31.44 nm) and the peaks matched with JCPDS Card #: ICSD ID 00-022-1189. The plane of three clear peaks and interplanar spacing ‘d’ were (003) at 0.241nm, (012) at 0.2088nm and (104) at 0.1477 nm (Figure 1c). The XRD profile displayed a rhombohedral shape of synthesised NiO particles. The intense and sharp peaks exposed that NiONPs were successfully shaped in the stem broth of *B. balochistanica.*

**Figure 2:**
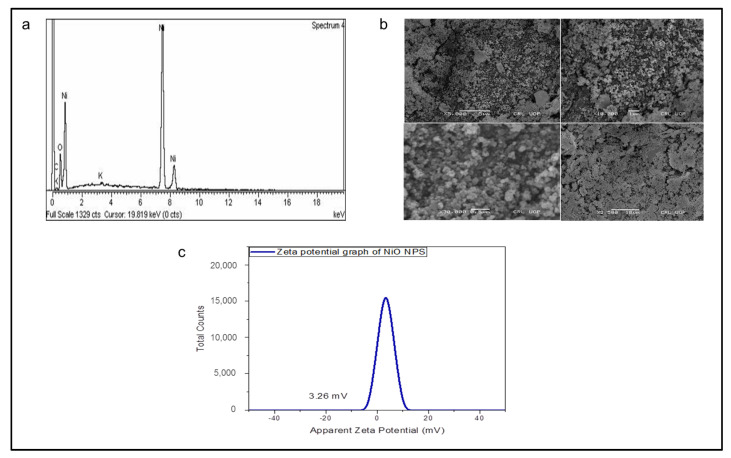
Spectroscopic and microscopic profile of BBS-NiONPs. (**a**) Energy dispersion spectroscopy (EDS) profile shows the elemental composition of BBS-NiONPs. Peaks are observed for Ni, O, C and K. (**b**) Micrograph of SEM shows the crystalline structure of BBS-NiONPs. (**c**) Zeta potential of BBS-NiONPs.

The elemental composition of synthesised nanoparticles was confirmed by EDS analysis. Figure 2a shows strong peaks of Ni (79.19%) and O (15.56%) by weight. The presence of carbon (4.50%) and potassium (0.74%) in graphs was attributed to grid support. Additionally, the morphological features of greenly invented BBS-NiONPs were assessed by scanning electron microscopy (SEM). Figure 2b illustrates the SEM profile of BBS-NiONPs confirming the highly agglomerated shape of the synthesised particles. The movement in suspension, for BBS-NiONPs, was studied by zeta-potential which was observed as 3.26 mV (Figure 2c).

### 2.2. Phytochemical and Antioxidant Analysis

**Figure 3:**
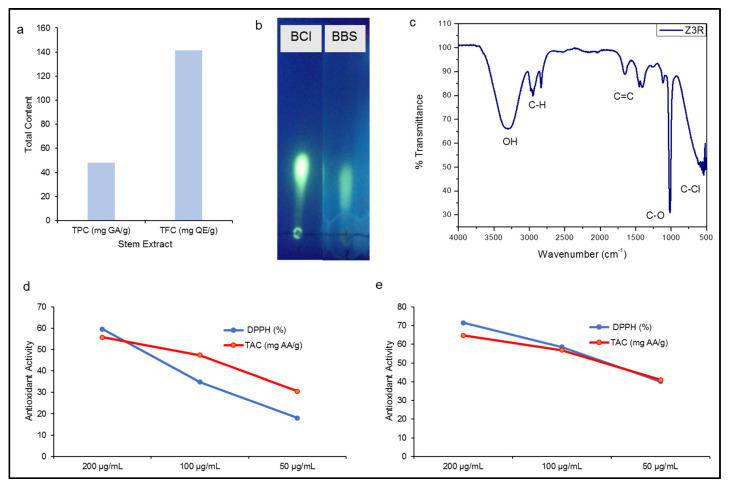
Phytochemical and Antioxidant analysis. (**a**) Total phenolic (mg GAE/g) and total flavonoid (mg QE/g) contents of stem extract. (**b**) Berberine in stem extract. BCl, berberine chloride; BBS, *B. balochistanica* stem. (**c**) FTIR spectrum of stem extract. (**d**) Antioxidant activities (DPPH and TAC) of stem extract. (**e**) Antioxidant activities (DPPH and TAC) of BBS-NiONPs. Numerical data are presented as mean ± standard deviation (*n* = 3).

In the present study, the stem extract of *B. balochistanica* plant was first time analysed for total phenolic contents (TPC) and total flavonoid contents (TFC) (Figure 3a). The concentrations of TPC and TFC were 48.21 mg GA/g and 141.29 mg QE/g. The presence of an adequate amount of berberine compound was also observed using thin-layer chromatography (TLC; Figure 3b). Additionally, the presence of biological molecules in stem extract of BB was also confirmed by FTIR spectroscopy. The functional groups were separated on the basis of peak values. The spectrum profile, peak values and functional groups were compared with the IR standard chart as shown in Figure 3c and Table 1. The FTIR spectrum of BBS extract showed multiples peak values indicating the presence of phenols, alcohols, alkanes, alkenes, aromatic compounds, carboxylic acid and alkyl halides. Interestingly, the absence of peaks at 2220–2260 cm^−1^ indicated the absence of cyanide derivatives.

Furthermore, the stem extract and green synthesised BBS-NiONPs were investigated for antioxidant potential via 2, 2-diphenyl-1-picrylhydrazyl (DPPH) and total antioxidant capacity (TAC). At 200 µg/mL, both stem extract and BBS-NiONPs showed remarkable antioxidant activities with a percentage inhibition of 59.61 and 71.48% (DPPH) and 55.78 and 64.77% (TAC), respectively (Figure 3d,e). Because of the presence of these phytocompounds (TPC, TPC and berberine) and strong antioxidant activities, stem extract was selected as stabilising and capping agents for the synthesis of BBS-NiONPs in the current experiment.

### 2.3. Antibacterial and Antifungal Activity of Phytofabricated NiONPs

**Figure 4:**
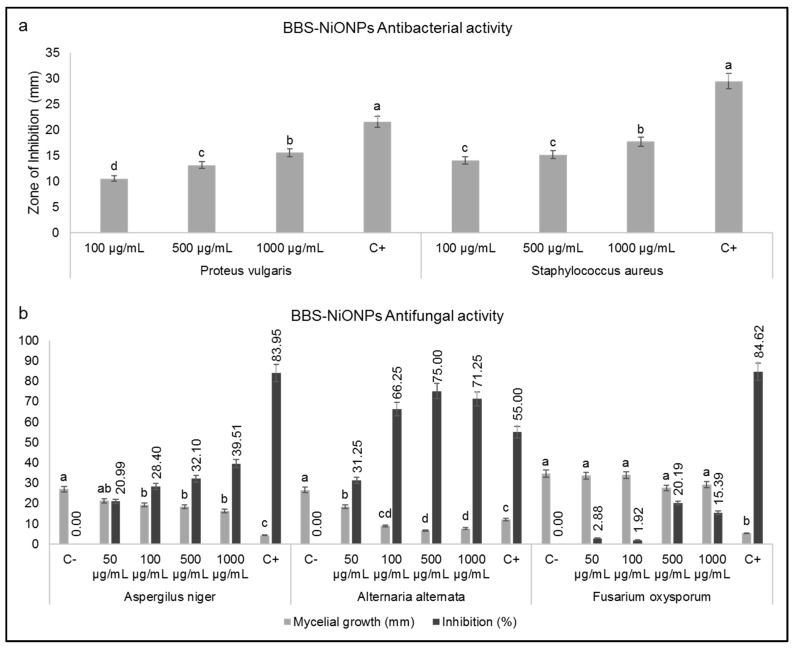
Antimicrobial potentials. (**a**) Antibacterial potential of BBS-NiONPs; C+ refers to Ciprofloxacin. (**b**) Antifungal potential of BBS-NiONPs. Letters indicate a significant difference (*p* < 0.05) between control and BBS-NiONPs treated samples. C+ refers to fluconazole. Numerical data are presented as mean ± standard deviation (*n* = 3).

Figure 4a exhibits the antibacterial activity of phytofabricated BBS-NiONPs (100, 500 and 1000 µg/mL) against *Proteus vulgaris* and *Staphylococcus aureus* bacterial strains. BBS-NiONPs showed a dose-dependent response against both selected bacterial strains. Moreover, 10 µg Ciprofloxacin (positive control) was found more reactive than all the applied doses of BBS-NiONPs during antibacterial activities.

Antifungal analysis of BBS-NiONPs was carried out using different concentrations (50, 100, 500, 1000 µg/mL) against *Fusarium oxysporum, Aspergillus niger* and *Alternaria alternata.* Dose-dependent responses against all three analysed fungus strains were observed (Figure 4b). *F. oxysporum* was found less vulnerable at high concentration (1000 µg/mL) while *A. alternata* was found highly susceptible with a percentage inhibition of 71.25% followed by *A. niger* with a percentage inhibition of 39.51% at 1000 µg/mL. 

### 2.4. Cytotoxic Potential of Biosynthesised BBS-NiONPs

**Figure 5:**
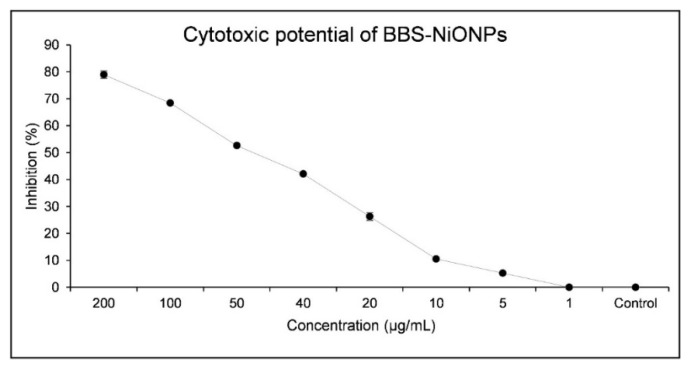
Brine shrimp mortality assays of BBS-NiONPs.

The cytotoxic potential of biosynthesised BBS-NiONPs was examined against brine shrimp (BS) larvae. The BS larvae were exposed to BBS-NiONPs at different concentrations (200-1 µg/mL) for 24 h and a considerable dose-dependent cytotoxic response with IC_50_ value (10.40 µg/mL) was calculated (Figure 5).

### 2.5. Inhibitory and Stimulatory Effect of BBS-NiONPs

**Figure 6:**
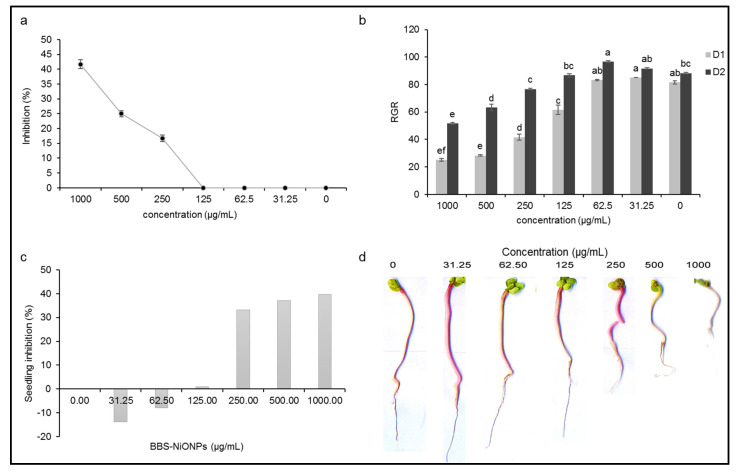
Effects of BBS-NiONPs on the seed germination and seedling growth of a radish plant. (**a**) Percentage inhibition (inhibitory effect) of seed germination against applied concentrations of BBS-NiONPs. (**b**) Relative germination rate (RGR) of seed (stimulatory effect) during first two days (D1 and D2). (**c**) Relative seedling growth rate (%). (**d**) Seedling length (mm) at applied concentrations. Letters indicate a significant difference (*p* < 0.05) between control and BBS-NiONPs treated samples. Numerical data are presented as mean ± standard deviation (*n* = 3).

Figure 6a signifies the percentage inhibition of seed germination at the applied concentration of BBS-NiONPs (31.25–1000 µg/mL). The percentage inhibition was observed in a dose-dependent manner. At lower concentrations (31.25–125 µg/mL), the seed germination was not inhibited, while at higher concentrations, the seed germination was inhibited as 16, 25 and 41% at 250, 500 and 1000 µg/mL, respectively. Furthermore, the relative germination rate (RGR) of seeds during the first two days was also more fascinating as at lower doses the germination started earlier in non-treated control. The seeds treated with BBS-NiONPs (31.25 and 62.5 µg/mL) showed 2–4% more germination than control (non-treated) after Day 1. After Day 2, the treated seeds (31.25 and 62.5 µg/mL) showed 3–8% stimulatory activity as compared to control (Figure 6b). In short, BBS-NiONPs showed stimulatory effects at lower doses and enhanced the speed of germination as compared to control (non-treated). Similarly, NiONPs also showed positive effects on the seedling growth of the radish plant. At lower concentrations (31.25 and 62.5 µg/mL), the seedling growth was improved by 13.86 and 7.92%, while at higher concentrations, the growth of the seedling declined by 33.17, 37.13 and 39.60% at 250–1000 µg/mL, respectively (Figure 6c,d).

## 3. Discussion

In *B. balochistanica* roots, many bioactive compounds such as phenolics, pakistanamine, proaphorphin, benzylisoquinoline, alkaloid, flavanols, berberine, oleanolic acid and gallic acid have been reported [13,14]. In the present study, the phytochemistry of BBS was investigated and remarkable antioxidant activities were noted. FTIR of BBS indicated the presence of multiple functional groups including phenols, alcohols, alkanes, alkenes, aromatic compounds, carboxylic acid and alkyl halides. These functional groups represent secondary metabolites that act as a natural defense system and give medicinal properties to plants [19]. 

Based on the results of the present study on phytochemicals with potent antioxidant activities, the BBS extract was selected to use in the green synthesis of NiONPs. Previously, NiONPs have been synthesised by physicochemical and biological methods [20]. However, the biocompatibility and phytochemical potential of BBS-NiONPs indicate that utilising medicinal plants for green synthesis is a much better strategy to synthesise pure, safe, biocompatible and bioactive NiONPs. Although using medicinal plants for green synthesis provides rather effective NPs [11], it poses a threat to biodiversity due to the fact that many medicinal plants, such as *B. balochistanica* used in this study, are subject to extreme pressure due to their extensive use by local and homeopathic communities. Unlike Dangi et al. where they used *B. asiatica* roots for green synthesis [11], we synthesised NiONPs using *B. balochistanica* stem extract and found compatible, or even better, bioactivities of the synthesised NiONPs. These findings encourage using aerial parts of medicinal plants in green synthesis and other pharmacological fields—an approach that can help in the preservation of rare species.

Colour observation, spectroscopic and microscopic analysis characterised the biogenic BBS-NiONPs. The formation of BBS-NiONPs was verified by changing the colour from green to greenish-gray. The UV-Vis spectra at 305 nm, average size 31.44 nm, pure Ni and O elemental nature and rhombohedral agglomerated shape clearly validated the formation of BBS-NiONPs. Additionally, the multiple bands corroborate the presence of biomolecules on the surface of BBS-NiONPs which act as capping agents and the peak at 616.36 cm^−1^ is linked with Ni-O bond formation. These results show uniformity with previous reports [21,22,23,24]. The BBS-NiONPs showed agglomerated shape which represented the electrostatic interaction of the synthesised NPs with each other. It is reported that this happens due to the nano size, high surface tension, surface energy and different reducing phytochemicals in different plant extracts [25,26]. Due to this agglomeration, the dispersity of BBS-NiONPs became low in suspension. 

The emerging resistance against antibiotics and evolving of new infectious microbial species are the main challenges for researchers. Therefore, the researchers are trying hard to develop nanomaterials with potent biological potentials against degenerative infectious diseases. The biogenic BBS-NiONPs showed astonishing DPPH radical scavenging and TAC antioxidant activities in a dose-dependent manner. As compared to BBS extract, BBS-NiONPs showed better antioxidant potential at all applied concentrations. Therefore, it is reflected that strong antioxidant activities of BBS-NiONPs are due to the interaction and adsorption of antioxidant compounds from the extract onto the surface of synthesised NPs [27]. Both bacterial isolates (gram-positive and gram-negative) were found susceptible to BBS-NiONPs. It coincides with the fact that NPs have the ability to penetrate inside the bacterial cell and obstruct metabolic activities [28]. BBS-NiONPs also showed antifungal response against tested fungal species. They inhibited mycelial growth in the following manner: *A. alternata* > *A. niger* > *F. oxysporum*. The BBS-NiONPs penetrate and enhance the permeability and generate reactive oxygen species (ROS) inside the fungal cell which retards mycelial growth [29]. The brine shrimp larvae motile movement was restricted by increasing the doses of BBS-NiONPs and ultimately mortality occurred within 24 h. This retardation of larval movement might be due to the attachment of NPs and when the NPs penetrated inside the larval body, they reduced the metabolic activity and, as a result, mortality occurred [30]. The dose-dependent mortality rate investigations have shown that the NPs can be used as anticancer drugs in biomedical fields [22,27,31]. The remarkable antioxidant, antibacterial, antifungal and cytotoxic potentials of BBS-NiONPs might be due to their nano gage dimension, precise surface area and adherence properties.

Due to the nano size, the BBS-NiONPs displayed potent biological activities against various infectious pathogens in the current study. NPs have more attachment and penetration ability with the cell membrane of pathogens as compared to bulk materials [29]. The exact mechanism of inhibitory activity of BBS-NiONPs is not clear. However, recent studies show that this inhibitory potential of NPs is due to the penetration and interference of NiONPs with intracellular machinery. Briefly, the BBS-NiONPs released nickel ions which attached and penetrated inside the cell and caused leakage of the cell membrane. Inside the cell, the NiONPs generated ROS, which directly inhibited the cellular life machinery like breaking phosphate and hydrogen bonding of the DNA strand, destroying the three-dimensional structure of proteins and causing oxidative stress in the power house of the cell [32].

Recently, NPs have also been used as biofertilisers in the agricultural field for enhancing nutrients uptake, breaking seed dormancy and reducing the application of hazardous agrochemicals [33]. Therefore, green synthesis of NPs might be useful for controlled release of fertiliser, plant growth mediation and green alternative to agrochemicals. Numerous metal NPs such as TiO_2_, ZnO and AgNPs are used as biostimulators in the agricultural field [34]. Significant reports are available on inhibitory potentials of NiONPs, while regarding stimulatory activities, little information is available [35,36]. In the present study, BBS-NiONPs increased the seed germination (2–8%) as compared to non-treated control, while at lower concentrations, the BBS-NiONPs showed nontoxic effects on seedling growth. In short, at a lower quantity, these green synthesised BBS-NiONPs are biocompatible and have the ability to speed up seed germination by breaking seed dormancy and can act as a growth-promoting agent. The positive response of seeds toward lower concentrations of BBS-NiONPs might be due to various factors such as infiltration of NPs, releasing ions and making a suitable environment for oxygen and water uptake, hence breaking seed dormancy and promoting seedling growth [33,37]. These findings indicate that apart from their potential in the biomedical field, green synthesised NPs could be effectively used in agricultural fields as nano fertilisers as well as stimulatory agents for plant physiology.

## 4. Materials and Methods

### 4.1. Collection of Berberis balochistanica Plant 

*B. balochistanica* was collected during June-August 2019 from mountainous regions of Hanna Urak, Quetta, Baluchistan, Pakistan (30°16′ 28.45″ N, 67°10′ 50.43″ E). The specimen of the collected plant was identified by comparing it with already present herbarium specimens and flora of Pakistan. The voucher specimen (RAW100268) was deposited in the National Herbarium, Islamabad, Pakistan for reference study.

### 4.2. Preparation of B. balochistanica Stem Extracts

The BBS was washed and shifted to the oven for 10 h at 40 °C. Later, the crushed fine powder of the stem (20.66 g) was thoroughly mixed with 200 mL of distilled water. After stirring for 12 h, the BBS extract was incubated in a water bath at 40 °C for 2 h. The prepared extract was filtered three times using Whatman filter paper and centrifuged at 3000 rpm for 30 m to remove the remaining aggregates. For experimental analysis, the stock solution of BBS was stored at 4 °C. Primarily, we examined the phytochemical and antioxidant potential of BBS extract and then used it as a stabilising and reducing agent in the green synthesis of BBS-NiONPs.

### 4.3. Phytochemical Analysis of BBS

#### 4.3.1. Berberine Analysis in BBS Extract 

The presence of berberine in BBS extract was screened by TLC, 10 µL of Berberine chloride (10 µg/mL) was used as a reference compound. A small drop of BBS extract and the reference drug were drawn using a capillary tube on a preactivated TLC plate (8 × 8 cm). The TLC plate was put into the TLC tank having a mobile phase (methanol:ethyl acetate:acetic acid:water) (5:4:1:1, *v/v*). After separation, the TLC plate was dried and visualised by UV-Vis spectrum at 365 nm. 

#### 4.3.2. Total Phenolics and Total Flavonoids Contents Analysis

The TPC was determined in stem extract using Folin-Ciocalteu reagent [38]. In brief, 20 µL of root extract was mixed with 90 µL of Folin-Ciocalteu reagent, and then with 90 µL of NaCO_3_ solution. After incubation at room temperature for 60 m, absorbance was measured. TPC was expressed as Gallic acid equivalents (mg GAE/g) of the sample. TFC was estimated using the Aluminium Chloride Colorimetric method with some modifications. TFC in stem extract was expressed as Quercetin equivalents (mg of QE/g) of the extract. 

### 4.4. Green Synthesis and Physical Characterisation of BBS-NiONPs

#### 4.4.1. BBS-NiONPs Synthesis

For the green synthesis of BBS-NiONPs, formerly used protocols with slight changes were used [27]. To synthesise BBS-NiONPs, 50 mL purified BBS extract solution was steadily added to the solution of NiNO_3_ (0.3 M). The mixture was subjected to heating at 60 °C with proper stirring at 500 rpm for 3 h. The precipitated pellet of BBS-NiONPs was collected after centrifugation at 3000 rpm for 25 m and washed with distilled water (three times). The presumptuous pellet of BBS-NiONPs was incubated at 100 °C to entirely evaporate the remaining water. Finally, the synthesised NiONPs were physically and biologically characterised using different techniques.

#### 4.4.2. Characterisation of BBS-NiONPs 

The bio-reduction of NiNO_3_ into NiONPs was confirmed by the colour changing and optical properties of this reduced solution were inveterate via absorption spectra using UV-400 UV-Vis spectrophotometer (Germany) at a scanning range of 200 to 700 nm. FTIR at scanning range 400–4000 cm^−1^ was used to verify the capping and stabilising properties of various functional groups associated with the green synthesis of NPs. Additionally, the structural, elemental, vibrational and morphological nature of BBS-NiONPs were studied using XRD, Raman spectroscopy and SEM (NOVA FEISEM-450 fortified with EDX apparatus). The size of biosynthesised NiONPs was calculated by the Scherrer equation.

D=k λ/βcosθ

### 4.5. Antioxidant Activity of BBS and BBS-NiONPs

#### 4.5.1. DPPH (2,2-Diphenyl-1-picrylhydrazyl) Assay

In this method, the in vitro free radical-scavenging potential of BBS and BBS-NiONPs at different concentrations (50, 100 and 200 µg/mL) was determined using a microplate reader [39]. Reagent solution was prepared by adding 2.4 mg of DPPH to 25 mL of methanol. The procedure involved the addition of 180 µL of reagent solution into 20 µL of the test sample to make 200 µL of the final reaction mixture. The mixture was subjected to a shaker followed by incubation for 1 h. Ascorbic acid was used as a reference antioxidant. DPPH solution without sample was taken as control and methanol was used as a blank solution. Finally, the absorbance of the control and tested samples was measured at 517 nm using a microplate reader to find radical scavenging activity using the following formula below:

$$\mathrm{DPPH}\;\mathrm{scavenging}\;\mathrm{effect}\;\%=\;\frac{\mathrm{AC}-\mathrm{AN}}{\mathrm{AC}}\times 100$$

where, AC and AN refer to the absorbance of the control and NPs at 517 nm.

#### 4.5.2. Total Antioxidant Capacity

The TAC of BBS and BBS-NiONPs was evaluated by the phosphomolybdenum method [40]. NiONPs and the reagent solution (0.6 mol/L sulfuric acid, 28 mmol/L sodium phosphate and 4 mmol/L ammonium molybdate) were mixed and incubated at 95 °C for 90 m. The solution was cooled, and the absorbance of the mixture was taken at 695 nm. TAC was calculated as μg/mg equivalent of ascorbic acid (µg AA/mg). 

### 4.6. Anti-Microbial Analysis of BBS-NiONPs

#### 4.6.1. Antibacterial Screening Using Disc Diffusion Method (DDM)

The disc diffusion method was used for antibacterial screening of green synthesised BBS-NiONPs against gram-negative (*P. vulgaris*) and gram-positive strains (*S. aureus*). Bacterial strains used in the present study were obtained from the culture bank (hospital isolates) of the Microbiology Laboratory, Faculty of Biological Science, Quaid-i-Azam University, Islamabad, Pakistan. Both strains were characterised by biochemical and cultural assessment [41]. Firstly, the Muller-Hinton agar was prepared, autoclaved and finally, the cooled media was poured into Petri plates. After solidifying, the bacterial strains were streaked out and a paper disc holding different concentrations of BBS-NiONPs (100, 500, 1000 µg/mL) and 10 µL of Ciprofloxacin (positive control) were put on bacterial cultures. The prepared plates were incubated at 37 °C overnight and bactericidal activity of plant-made BBS-NiONPs was observed for the zone of inhibition (mm) around the coated discs.

#### 4.6.2. Antifungal Assay Using Poisoned Food Method (PFM)

The poisoned food method (PFM) was used to study the antifungal activity of BBS-NiONPs using different phytopathogenic fungal pathogens (*A. niger*, *A. alternata* and *F. oxysporum*). All fungal pathogens were obtained from Plant Pathology Laboratory, Department of Plant Sciences, Quaid-i-Azam University, Islamabad, Pakistan [29]. These fungal pathogens were cultured using autoclaved Sabouraud dextrose agar media (SDA; Oxoid CMO147). Different doses (100, 500 and 1000 µg/mL) of prepared BBS-NiONPs solution were mixed with SDA media, shaken properly, and poured into Petri plates for solidification. A disc of 7 days old fungal culture (5 mm) was put in the middle of the media plates. The non-treated SDA media and fluconazole-treated SDA media were used as the negative and positive controls, correspondingly. After incubation for 3 days at 27 °C, the mycelial growth against various concentrations of BBS-NiONPs was measured in millimetres and the percentage of inhibition was calculated by the given formula [42].

$$\mathrm{Percentage}\;\mathrm{Inhibition}=\mathrm{FC}-\frac{\mathrm{FN}}{\mathrm{FC}}\times 100$$

where, FC and FN represent the average increase in fungal growth (F) in the control and each treatment (NPs).

### 4.7. Cytotoxic Assay of BBS-NiONPs by PFM

#### 4.7.1. Brine Shrimp Cytotoxicity Assay (BSCA) of BBS-NiONPs

The cytotoxic effects of BBS-NiONPs were evaluated using the brine shrimp cytotoxicity assay. For this purpose, artificial seawater was prepared by using 3.8 g sea salt in 1 L distilled water in a hatching chamber having a partition. Eggs of brine shrimp (*Artemia salina*) were put in a covered portion of the chamber and incubated for 48 h at 30 °C. After hatching, 20 mature brine shrimp larvae were shifted to individual glass vials having various amounts of BBS-NiONPs (200-1 µg/mL) and the final volume was adjusted up to 5 mL by adding seawater. After 24 h, alive brine shrimps were counted [43]. Lethality concentration (LC_50_ values) and percentage mortality were calculated using GraphPad software. 

#### 4.7.2. Phytotoxicity Assay of BBS-NiONPs

The phytotoxic effect of green-made NPs was evaluated using the radish seed assay method (RSA) [44]. Different concentrations (31.25–1000 µg/mL) of BBS-NiONPs solution were introduced in each Petri plate containing sterilised filter paper (Whatman filter paper) and 15 seeds. The obtained data was measured as mean ± standard deviation (*n* = 3) and different seed germination indices (final germination, percentage inhibition) were calculated [45]. Finally, the seedling length was measured in mm.

### 4.8. Statistical Analysis

The obtained data were reported in triplicates and quantified as mean ± standard deviation. To check the inhibitory potential of BBS-NiONPs on bacterial, fungal and seedlings growth, a statistical valuation was performed by one-way analysis of variance. Multiple comparisons between means were estimated by LSD test at 95% confidence interval. All statistical analyses were performed using Statistix version 10. The cytotoxic activity of BBS-NiONPs was estimated by calculating the lethality concentration (IC_50_) values using the probit analysis (GraphPad software, San Diego, CA, USA) [46,47].

## 5. Conclusions 

The present study reports the green synthesis of BBS-NiONPs from the stem extract of the *Berberis balochistanica* plant. The presence of valuable phytochemicals with bioactive functional groups and potent antioxidants in stem extract helped in stabilising, capping and reducing nickel salt into BBS-NiONPs. The crystalline rhombohedral shape and fine size (31.44 nm) of BBS-NiONPs were confirmed by SEM and XRD techniques. Remarkable biological applications of BBS-NiONPs, like antioxidant, antimicrobial and cytotoxic potentials, were observed. BBS-NiONPs were also found as biostimulators in boosting up the germination frequency and seedling growth at suitable quantities. This indicates that they could be used as a substitute for synthetic chemicals in biomedical and agricultural fields. Moreover, they are also suitable for plants with high dormancy and slow seed germination. However, a further widespread investigation is recommended before introducing BBS-NiONPs into clinical and agricultural trials.

## Figures and Tables

**Table 1:** FTIR analysis of *B. balochistanica* plant stem extract.

Peak Values (cm^−1^)	Strength ^a^	Functional Groups	Interpretations
3306.95	Medium	OH	Phenol, Alcohol
2947.93–2834.58	Medium	C–H	Alkane
1655.05	Weak	C=C	Alkene
1449.31	Weak	C=C	Aromatic compounds
1114.57	Weak	C–O	carboxylic acids, alcohols
1016.4016	Strong	C–O	carboxylic acids, alcohols
587.88–522.90	Medium	C–Cl	Alkyl halides, Sulphur compounds

^a^ Strength of peaks in the spectrum (500–4000 cm^−1^).

## Data Availability

All data used for the present study has been provided within the manuscript. For further assistance, if required, please contact the corresponding author(s).
